# Differential Effects of the Six Human TAU Isoforms: Somatic Retention of 2N-TAU and Increased Microtubule Number Induced by 4R-TAU

**DOI:** 10.3389/fnins.2021.643115

**Published:** 2021-05-25

**Authors:** Sarah Bachmann, Michael Bell, Jennifer Klimek, Hans Zempel

**Affiliations:** ^1^Institute of Human Genetics, Faculty of Medicine and University Hospital Cologne, University of Cologne, Cologne, Germany; ^2^Center for Molecular Medicine Cologne (CMMC), Faculty of Medicine and University Hospital Cologne, University of Cologne, Cologne, Germany

**Keywords:** TAU, MAPT, microtubule-associated proteins, microtubule dynamics, primary neuron cell culture, SH-SY5Y cell line, axonal targeting, somatodendritic localization

## Abstract

In the adult human brain, six isoforms of the microtubule-associated protein TAU are expressed, which result from alternative splicing of exons 2, 3, and 10 of the *MAPT* gene. These isoforms differ in the number of N-terminal inserts (0N, 1N, 2N) and C-terminal repeat domains (3R or 4R) and are differentially expressed depending on the brain region and developmental stage. Although all TAU isoforms can aggregate and form neurofibrillary tangles, some tauopathies, such as Pick’s disease and progressive supranuclear palsy, are characterized by the accumulation of specific TAU isoforms. The influence of the individual TAU isoforms in a cellular context, however, is understudied. In this report, we investigated the subcellular localization of the human-specific TAU isoforms in primary mouse neurons and analyzed TAU isoform-specific effects on cell area and microtubule dynamics in human SH-SY5Y neuroblastoma cells. Our results show that 2N-TAU isoforms are particularly retained from axonal sorting and that axonal enrichment is independent of the number of repeat domains, but that the additional repeat domain of 4R-TAU isoforms results in a general reduction of cell size and an increase of microtubule counts in cells expressing these specific isoforms. Our study points out that individual TAU isoforms may influence microtubule dynamics differentially both by different sorting patterns and by direct effects on microtubule dynamics.

## Introduction

**FIGURE 1:**
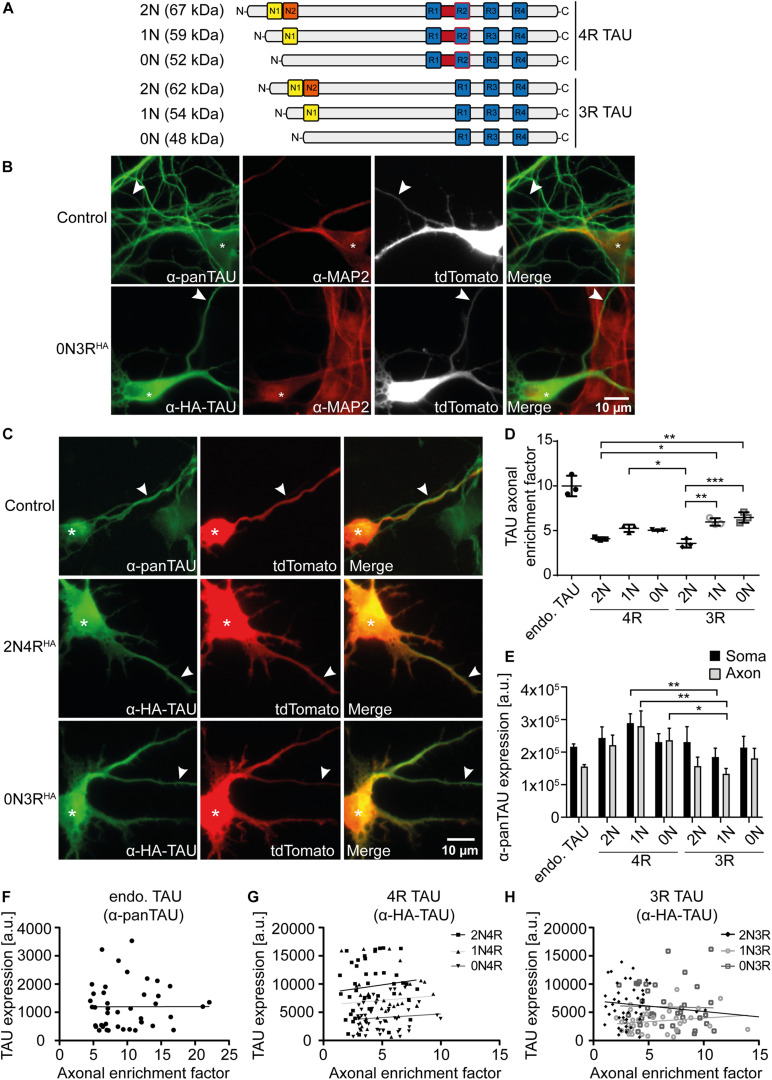
2N-containing TAU^HA^ isoforms are less efficiently sorted into the axon in mouse primary neurons. HA-tagged TAU isoforms (0N, 1N, 2N, and 3R or 4R) and tdTomato as a volume marker were co-transfected into mouse primary neurons (DIV4) and expressed for two days. **(A)** Schematic overview of the six TAU isoforms expressed in human brains and their estimated sizes in kDa. N1, N2 = N-terminal inserts; R1–R4 = C-terminal repeat domains. **(B)** Representative images of mouse primary neurons (DIV6) transfected with tdTomato (Ctrl.) or co-transfected with tdTomato and 0N3R-TAU, respectively. Neurons were stained with α-pan-TAU (for endogenous TAU in control) or α-HA antibody (for transfected TAU^HA^) and α-MAP2 to distinguish axons and dendrites. Arrowheads indicate axons, asterisks indicate cell bodies. **(C)** Representative images of mouse primary neurons (DIV6) transfected with tdTomato (Ctrl.) or co-transfected with tdTomato and 2N4R-TAU^HA^ and 0N3R-TAU^HA^, respectively. Neurons were stained with α-Tau (for control) or α-HA antibody to analyze axonal enrichment of transfected or endogenous TAU. Arrowheads indicate axons, asterisks indicate cell bodies. **(D)** Axonal enrichment of TAU was calculated from soma-to-axon ratio of TAU fluorescence intensity and normalized to soma-to-axon ratio of the tdTomato signal. An axonal enrichment of one is considered as a random distribution. *N* = 3, at least 10 cells were analyzed per condition. Error bars represent SEM. Shapiro–Wilk test was performed to test for normal distribution of data; statistical analysis was performed by one-way ANOVA with Tukey’s test for correction of multiple comparisons. Statistical significance: *p ≤ 0.05; **p ≤ 0.01; ***p ≤ 0.001. **(E)** Comparison of pan-TAU expression in axon and soma of untransfected neurons (endo.TAU) and TAU^HA^ isoform-expressing neurons. Error bars represent SEM. Shapiro–Wilk test was performed to test for normal distribution of data; statistical analysis was performed by Kruskal–Wallis test with Dunn’s correction for multiple comparisons. Statistical significance: *p ≤ 0.05; **p ≤ 0.01. **(F–H)** Linear regression of somatic TAU expression and axonal enrichment. Linear regression was performed for **(F)** untransfected neurons (endo. TAU), **(G)** 4R isoform-expressing neurons, and **(H)** 3R isoform-expressing neurons. No significant correlation of TAU expression levels and axonal enrichment was observed for all experimental groups.

The human microtubule-associated protein TAU is encoded by the *MAPT* gene on chromosome 17. Expression of *MAPT* results in six major TAU isoforms in the adult human central nervous system and two isoforms in the peripheral nervous system (Goedert et al., 1989, 1992; Andreadis et al., 1992; Couchie et al., 1992). The brain-specific isoforms vary in the number of N-terminal inserts (0N, 1N, or 2N) and C-terminal repeat domains (3R or 4R) due to alternative splicing of exons 2, 3, and 10, resulting in sizes between 48 kDa (0N3R) and 67 kDa (2N4R) of the corresponding proteins (Goedert et al., 1989; Figure 1A). TAU isoform expression is directly linked to brain development: During neurogenesis, only the shortest TAU isoform, 0N3R, is expressed, whereas in the adult brain, all six isoforms are present with roughly equal amounts of 3R and 4R isoforms (Goedert et al., 1989; Trabzuni et al., 2012). Splicing of TAU is also species-dependent, e.g., TAU isoform expression in rodents shifts from 0N3R during brain maturation to only 4R isoforms in adults (McMillan et al., 2008; Bullmann et al., 2009). Accumulation of TAU in neurofibrillary tangles (NFTs) is a hallmark of many neurodegenerative diseases, named tauopathies. All isoforms are potent to form NFTs under pathological conditions; causes can be mutations in *MAPT* affecting splicing or function of TAU, or mislocalization of TAU into the somatodendritic compartment upon cellular stress (reviewed in Arendt et al., 2016). Tauopathies can be classified *via* the isoforms that accumulate in NFTs: While TAU tangles mainly consist of 3R-TAU isoforms, e.g., in Pick’s disease (PiD) and 4R-TAU in progressive supranuclear palsy (PSP), both 3R- and 4R-TAU isoforms are present in NFTs of Alzheimer’s disease patients (Goedert et al., 1995; Buee and Delacourte, 1999; Arai et al., 2003). During brain development and especially during neuronal polarization, TAU becomes efficiently sorted into the axon (Mandell and Banker, 1995). In the adult human brain, TAU is mainly localized in the axon; however, a small fraction can also be observed in the somatodendritic compartment and in the nucleus (Binder et al., 1985; Rady et al., 1995; Tashiro et al., 1997). The subcellular distribution of TAU seems to be isoform-specific, e.g., 2N isoforms show a higher propensity for a somatodendritic localization than other variants (Zempel et al., 2017a). Axonal targeting of TAU is thought to be mediated by a variety of processes, such as the presence of a TAU diffusion barrier (TDB) at the axon initial segment (AIS), which prevents retrograde diffusion of TAU (Li et al., 2011; Zempel et al., 2017a). Furthermore, microtubule-binding affinity of TAU might be higher in the axon, likely accomplished by the presence or absence of posttranslational modifications (PTMs), such as phosphorylation and acetylation (Evans et al., 2000; Kishi et al., 2005; Tsushima et al., 2015). TAU interactions are also important for proper sorting of TAU., e.g., interaction with the calcium-regulated plasma membrane-binding protein Annexin A2 was shown to link microtubules and the membrane of the growth cone, thereby trapping TAU at the presynaptic membrane (Gauthier-Kemper et al., 2011). Through its interaction with microtubules, TAU supports axonal differentiation, morphogenesis, outgrowth, transport, and neuronal plasticity (Esmaeli-Azad et al., 1994; Kempf et al., 1996; Takei et al., 2000). *In vitro* studies already described a reduced microtubule-binding affinity and assembly for 3R-TAU isoforms (Goedert and Jakes, 1990; Goode et al., 2000; Panda et al., 2003), which is in line with the fact that the C-terminal repeat domains together with the proline-rich linker domain mediate microtubule binding (Goode et al., 1997). If and how the different TAU isoforms alter microtubule dynamics a *in vivo* is still unclear and might also depend on the differential subcellular localization of the isoforms. In this study, we address these questions by using primary mouse neurons and SH-SY5Y human neuroblastoma cells as neuronal model systems. Our results show that human TAU isoforms differ in their subcellular localization and that especially 2N-TAU isoforms are less axonally enriched than shorter isoforms. In addition, we show an isoform-specific effect on cell size and microtubule number: Expression of 4R-TAU isoforms, for example, results in smaller cells and increased microtubule counts. Microtubule dynamics, such as microtubule stability, run length, and growth rate, are not altered upon expression of different TAU isoforms in undifferentiated SH-SY5Y cells. We show here that individual TAU isoforms may influence microtubule dynamics differentially both by different sorting patterns as well as by direct effects on microtubule dynamics in a non-disease background.

## Methods

### Cell Culture

SH-SY5Y cells were cultured in DMEM/F12, GlutaMAX [Thermo Fisher Scientific (TFS)] supplemented with 10% fetal bovine serum (FBS, Biochrom AG), and 1 × antibiotic/antimycotic solution (TFS) in a humidified incubator at 37°C, 5% CO_2_.

Primary mouse neurons were isolated and cultured as described before (Zempel and Mandelkow, 2017) with slight modifications. In brief, brains of FVB/N mouse embryos were dissected at embryonic day 13.5. Brainstem and meninges were removed and the whole cortex was digested with 1 × trypsin (PAN-Biotech). The cell suspension was diluted in prewarmed (37°C) neuronal plating medium [Neurobasal media (TFS), 1% FBS, 1 × antibiotic/antimycotic solution (TFS), 1 × NS21 (PAN-Biotech)] seeded onto coated plates, and cultivated in a humidified incubator at 37°C, 5% CO_2_. Four days after plating, the medium was doubled with neuronal maintenance media [Neurobasal media (TFS), 1 × antibiotic/antimycotic solution (TFS), 1 × NS21 (PAN-Biotech)], and cells were treated with 0.5 μg/ml AraC (Sigma-Aldrich).

### Microtubule Dynamics

SH-SY5Y cells were co-transfected with expression plasmids containing Dendra2c-TAU (TAU^*D*2^) isoforms and tdTomato-N1-EB3 by using Lipofectamine2000 (TFS) according to the manufacturer’s protocol. Control cells were transfected with tdTomato-N1-EB3 and an empty Dendra2c plasmid. Only cells showing both Dendra2c and tdTomato signal were used for analysis. Two days after transfection, cells were transferred into a live-cell imaging chamber (ALA Scientific) and EB3 comets of a single cell were imaged for 120 s (1 frame per second) with a Leica DMi8 microscope (Leica). The original movie file was processed by ImageJ software (Schindelin et al., 2012; Schneider et al., 2012) as follows: An average minimum projection was calculated from all frames and subsequently subtracted from all. Afterwards, a threshold was set and microtubule dynamics were analyzed by ImageJ plugin TrackMate (Tinevez et al., 2017), using LoG detector with an estimated blob size of 2 px. The following parameters were examined: cell area (μm^2^), microtubule number/μm^2^, microtubule run length (μm), microtubule stability (s), and microtubule growth rate (μm/s). Data were filtered for quality ≥1.5, track start >0 s, and track end <120 s. All experiments were conducted in three replicates and at least five cells were analyzed per condition. Cell area was measured from at least 30 cells. Shapiro–Wilk test was performed to test for normal distribution of data; afterwards, statistical analysis was performed by either one-way ANOVA with correction for multiple comparisons (Tukey’s test) for normally distributed data or Kruskal–Wallis test with Dunn’s correction for multiple comparison of non-parametric data using GraphPad Prism software (v8.0.1). The statistical test performed is highlighted in the figure legends for each graph.

### Cell Lysis and Western Blot Analysis

SH-SY5Y cells were lysed with RIPA buffer [50 mM HEPES pH 7.6, 150 mM NaCl, 1 mM EDTA, 1% Triton X-100, 0.1% sodium dodecyl sulfate, 0.5% sodium deoxycholate, 1 × cOmplete ULTRA EDTA-free protease inhibitor cocktail (Merck)]. Samples were diluted with SDS buffer and separated on 10% polyacrylamide gels. Proteins were transferred to PVDF membranes and blocked in 5% non-fat dry milk in TBS-T. Membranes were incubated with the primary antibody overnight at 4°C, washed with TBS-T, and incubated with the corresponding secondary HRP-coupled antibody for one hour at room temperature. The used antibodies are listed in Table 1. Luminescent signals were detected with ChemiDoc XRS + system (Bio-Rad).

**TABLE 1:** Antibodies used in this study.

**Antibody**	**Company**	**Cat. no.**	**Application**	**Dilution**
**Primary antibodies**				
Rabbit polyclonal anti-TAU (K9JA)	Dako	A0024	WB / ICC	1:10,000 / 1:1,000
Chicken polyclonal anti-MAP2	Abcam	ab5392	ICC	1:2,000
Mouse monoclonal anti-HA (16B12)	Biolegend	901501	ICC	1:1,000
Mouse monoclonal anti-GAPDH (1D4)	Novus Biologicals	NB300-221	WB	1:2,000
**Secondary antibodies**				
Donkey anti-mouse IgG, Alexa Fluor 488	Thermo Fisher Scientific	A21202	ICC	1:1,000
Goat anti-chicken IgG, Alexa Fluor 647	Thermo Fisher Scientific	A21449	ICC	1:1,000
Donkey anti-rabbit IgG, Alexa Fluor 488	Thermo Fisher Scientific	A21206	ICC	1:1,000
Phalloidin, Alexa Fluor 568	Thermo Fisher Scientific	A12380	ICC	1:40
Goat anti-mouse IgG (H + L), HRP-linked	Dianova	115-035-003	WB	1:20,000
Goat anti-rabbit IgG, HRP-linked	Cell Signaling Technologies	7074	WB	1:200,000

### Axonal Enrichment Factor and TAU Expression

To analyze TAU axonal enrichment, primary mouse neurons were transfected as described before with Lipofectamine2000 (TFS) (Zempel et al., 2017b). In brief, neurons were co-transfected with the corresponding N-terminally HA-tagged TAU (TAU^HA^) isoform and tdTomato. After two days, neurons were fixed and stained for endogenous mouse Tau with a pan-TAU antibody, or HA for exogenous TAU^HA^ and MAP2 as described before (Zempel and Mandelkow, 2017), using antibodies listed in Table 1. After imaging on a fluorescence microscope (Axioscope 5, Zeiss), axonal sorting was analyzed by measuring mean fluorescence intensities (MFI) of tdTomato and TAU in the axon and soma. Axons were identified by the absence of MAP2 and the presence of TAU signal, branching pattern of ∼90°, a length of above 300 μm, and constant diameter. Axonal enrichment factor (AEF) was calculated as follows: AEF = (MFI_(TAU,Axon)_/MFI_(TAU,Soma)_)/(MFI_(tdTom,Axon)_/ MFI_(tdTom,Soma)_). The experiment was performed in three replicates and at least 10 cells were analyzed per condition. For the analysis of TAU expression, neurons were transfected with TAU^HA^ isoforms, fixed, and stained for TAU by using a pan-TAU antibody and HA-tagged TAU by using an anti-HA antibody (see Table 1 for details). Expression was analyzed by measuring the MFI of pan-TAU in the soma and axon of untransfected and transfected cells.

Shapiro–Wilk test was performed to test for normal distribution of data; afterwards, statistical analysis was performed by either one-way ANOVA with correction for multiple comparisons (Tukey’s test) for normally distributed data or Kruskal–Wallis test with Dunn’s correction for multiple comparison of non-parametric data using GraphPad Prism software (v8.0.1). The statistical test performed is highlighted in the figure legends for each graph.

### Antibody List

The antibodies used for this study are listed in Table 1.

## Results

### TAU Axonal Sorting Is Isoform-Specific and Independent of Expression Levels

TAU is considered an axonal protein in mature neurons (Binder et al., 1985). However, previous results indicate a difference in axonal enrichment of the six human TAU isoforms that were overexpressed as Dendra2c-tagged fusion proteins in primary rodent neurons (Zempel et al., 2017a). To further investigate TAU isoform axonal sorting and rule out the influence of a big protein tag (such as Dendra2c with approx. 26 kDa) on cellular localization, all six human TAU isoforms were fused to an HA-tag (TAU^HA^) and co-transfected for two days with a volume marker (tdTomato) into primary mouse neurons aged for 7–9 days (DIV). One benefit of the HA-tag is the relatively small size, which leads to a size shift of the TAU isoforms of only 1.1 kDa (Figure 1A). Control neurons were transfected only with tdTomato as a volume marker, assuming an unbiased axodendritic distribution after two days of expression. To quantify axonal targeting of the different versions of TAU, we normalized the axonal presence of TAU against the unbiased distribution of tdTomato, expressed as the AEF (see *Methods* for details). To distinguish axons and dendrites, neurons were fixed and stained for endogenous mouse TAU or transfected human TAU^HA^ using an anti-HA antibody and the dendritic marker MAP2. Axons were identified by the absence of MAP2, the presence of TAU or HA signal, and further morphological criteria (see *Methods* for details; Figure 1B). Differences in axonal sorting of TAU were observed for the different TAU^HA^ isoforms (Figures 1C,D). For endogenous TAU, which mostly consists of 0N3R in prenatal rodent brains (McMillan et al., 2008; Bullmann et al., 2009), a strong axonal localization is visible from the immunofluorescence images, indicated by a strong TAU signal in the axon and only low levels in the soma and dendrites (AEF of ∼10, Figures 1C,D). Strong axonal enrichment was also observed in neurons expressing 0N3R-TAU^HA^. In contrast, neurons expressing 2N4R-TAU^HA^ show lower levels of axonal TAU but still strong axonal targeting compared to tdTomato (Figure 1C). To compare the different TAU^HA^ isoforms quantitatively, axonal enrichment of TAU was calculated from soma-to-axon ratio of TAU fluorescence intensity and normalized to soma-to-axon ratio of the tdTomato signal (Figure 1D). Axonal enrichment of endogenous TAU was approximately 10-fold higher compared with tdTomato, while the human TAU^HA^ isoforms showed approximately 4- to 6-fold enrichment (Figure 1D). From all six transfected isoforms, 0N3R-TAU^HA^ showed the strongest axonal enrichment, which reaches approximately 65% of endogenous TAU. A relatively efficient (but still ∼40% less than endogenous TAU) axonal sorting could also be observed for 1N3R-TAU^HA^. Of note, both 2N-TAU^HA^ isoforms showed the weakest axonal enrichment. Differences in TAU expression levels were assessed by measuring mean fluorescence intensities of the pan-TAU signal in the soma and axon (MFI) of neurons expressing only endogenous TAU (endo. TAU) or neurons co-expressing endogenous TAU with TAU^HA^ (Figure 1E). Comparison of expression rates of endogenous TAU with neurons expressing endogenous and TAU^HA^ revealed only slight overexpression rates for the different TAU isoforms (up to 0.5 times), both in the soma and axon, which were not significantly higher than in control cells (Figure 1E). This might indicate that either primary neurons downregulate endogenous TAU expression to prevent overexpression of TAU or that the amount of exogenously expressed TAU is small compared with endogenous TAU. To rule out that differential expression and overexpression of TAU isoforms influences cellular sorting mechanisms, we correlated somatic TAU expression levels of endogenous TAU and TAU^HA^ with the axonal enrichment of TAU (Figures 1F–H). No significant correlation was observed for any of the experimental groups, indicating that the expression levels likely do not impact TAU sorting. Taken together, our results implicate that the efficiency of axonal sorting of TAU is independent of TAU expression levels. Furthermore, the subcellular distribution of TAU seems to be isoform dependent and is influenced by the number of N-terminal inserts, since 2N4R- and 2N3R-TAU^HA^ showed the lowest axonal enrichment. No significant difference was observed between the other 3R- and 4R-expressing neurons, hinting towards a repeat-independent sorting mechanism of the isoforms.

### Differential Effect of TAU Isoforms on Cell Size and Microtubule Number

**FIGURE 2:**
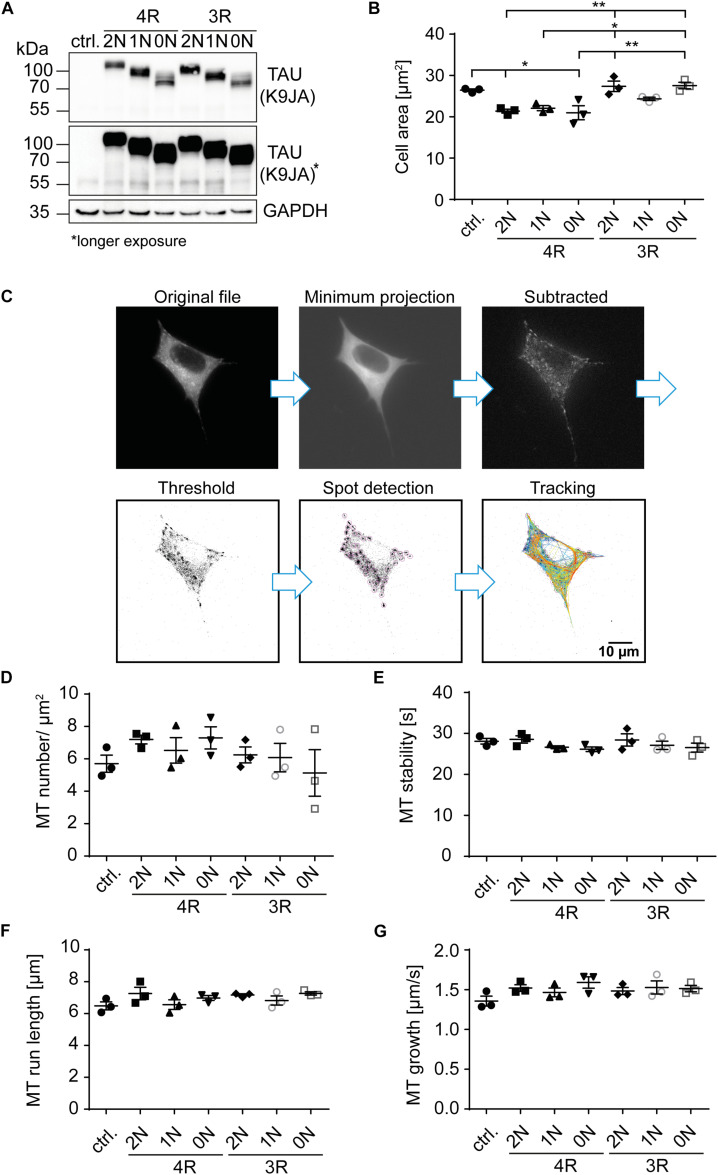
4R-TAU isoforms decrease cell size and increase microtubule counts in undifferentiated SH-SY5Y neuroblastoma cells. Expression of TAU^*D*2^ isoforms (0N, 1N, 2N, and 3R or 4R) in undifferentiated SH-SY5Y cells. **(A)** Western blot of SH-SY5Y cells transfected with the corresponding TAU^*D*2^ isoforms. Longer exposure of TAU signal shows negligible endogenous TAU expression (∼55 kDa). GAPDH was used as a loading control. **(B)** Cell area was measured from SH-SY5Y cells, co-transfected with tdTomato-N1-EB3 and TAU^*D*2^. Cell area was analyzed from at least 30 cells. Error bars represent SEM. Statistical analysis was performed by one-way ANOVA with Tukey’s test for correction of multiple comparisons. Asterisks indicate statistical significance: *p ≤ 0.05; **p ≤ 0.01. **(C)** Image processing for input into TrackMate. Minimum projection was calculated from all frames and subtracted from all. Afterward, threshold was set, and microtubule dynamics were analyzed by TrackMate. **(D–G)** Microtubule dynamics of SH-SY5Y cells co-transfected with Dendra2c-tagged TAU isoforms (0N, 1N, 2N, and 3R or 4R) and tdTomato-N1-EB3. Growing microtubule plus-ends were monitored in living cells for 2 min (1 fps). The following parameters were examined: **(D)** microtubule number (MT number/μm^2^) (normalized to corresponding cell area), **(E)** microtubule run length (μm), **(F)** microtubule stability (s), and **(G)** microtubule growth rate (μm/s). *N* = 3, at least five cells were analyzed per condition. Error bars represent SEM. Shapiro–Wilk test was performed to test for normal distribution of data; statistical analysis was performed by one-way ANOVA with Tukey’s test for correction of multiple comparisons for **(D–F)**. Kruskal–Wallis test with Dunn’s correction for multiple comparisons was performed for panel **(G)**.

One main function of TAU is its role in microtubule stability and spacing (Weingarten et al., 1975; Witman et al., 1976). The influence of individual TAU isoforms on microtubule dynamics, however, has not been addressed in living cells. To investigate TAU isoform-specific effects on microtubules, SH-SY5Y neuroblastoma cells were transfected with TAU^*D*2^ isoforms. The expression levels of TAU^*D*2^ were confirmed by Western blot analysis (Figure 2A). Of note, only low levels of endogenous TAU (∼55 kDa) were observed in control and transfected cells, which most likely corresponds to 0N3R-TAU that is mainly expressed by undifferentiated SH-SY5Y cells (Figures 1A, 2A, long exposure) (reviewed in Bell and Zempel, 2020). Exogenously expressed TAU isoforms show a shift in size of approx. 26 kDa due to the fused D2-tag (Figure 2A). TAU^*D*2^ overexpression rate was assessed by comparing the amount of endogenous and exogenous TAU and considering a transfection efficiency of ∼15–20% in SH-SY5Y cells, which resulted in a ∼50-fold overexpression. Since differentiated SH-SY5Y cells show an increase of about 12-fold of endogenous TAU expression, overexpression of D2-tagged TAU isoforms was normalized to expression in differentiated SH-SY5Y cells, which results in approx. four times higher final expression of exogenously expressed TAU.

Since microtubules are essential components of the cytoskeleton, initial analysis of TAU isoform-specific effects focused on changes in cell size, which may hint at cytoskeletal alterations. Cell area was significantly (∼20%) smaller in cells overexpressing 4R-TAU^*D*2^ isoforms compared with control cells (Figure 2B). In addition, cells expressing 2N3R- and 0N3R-TAU^*D*2^ had a significantly greater cell area than 4R-expressing cells (∼20%), comparable with untransfected cells, while 1N3R only showed a trend towards increased cell size compared with the 4R-TAU^*D*2^ isoform-expressing cells (∼15% bigger cell size). To further investigate the role of TAU isoforms in microtubule dynamics, SH-SY5Y cells were co-transfected with the corresponding TAU^*D*2^ isoform and tdTomato-N1-EB3 (Figures 2C–G). EB3 binds the growing microtubule plus-ends and can be used in live-cell imaging to track microtubule assembly (Stepanova et al., 2003). Expression of TAU^*D*2^ was confirmed *via* the green fluorescence of the D2-tag in live-cell imaging. EB3 comets were recorded for 2 min (1 fps), images were processed as described in Figure 2C, and microtubule dynamics were analyzed afterward using TrackMate plugin for ImageJ (Tinevez et al., 2017). The overall number of microtubules was quantified and normalized to the corresponding cell area (Figure 2D). Microtubule count was slightly increased compared with the control cells (5.5 MTs/μm^2^) in cells expressing 2N4R- and 0N4R-TAU^*D*2^ (7.2 and 7.3 MTs/μm^2^, respectively). In contrast, 3R-TAU^*D*2^ isoforms showed no difference in microtubule counts compared with control cells, despite a slight trend towards a reduced number of microtubules for 0N3R. Comparing 3R- and 4R-TAU^*D*2^ isoforms showed that microtubule numbers were significantly decreased for 0N3R compared to 4R-TAU^*D*2^-expressing cells (Figure 2D). To further investigate how microtubule counts might be affected by TAU^*D*2^ isoforms, microtubule stability, total microtubule run length, and microtubule growth rate (Figures 2E–G) were analyzed. We observed several tendencies that, however, did not reach statistical significance: 1N- and 0N-TAU^*D*2^ isoforms seem to cause a slight decrease in microtubule stability independent of the presence of the second N-terminal repeat (Figure 2E), and 2N4R-TAU^*D*2^ seems to increase microtubule run length (Figure 2F). Microtubule growth rate was slightly, but not significantly, increased for individual TAU isoform-overexpressing cells compared with control cells (Figure 2G), but it was significantly increased (*p* < 0.0143, unpaired *t*-test) when we compared all TAU isoform-expressing cells to control cells. All in all, the results indicate that TAU isoforms have a significant impact on cell size and show slight differences in microtubule counts and impact on microtubule growth rate, without significant differential effects on microtubule stability and run length in undifferentiated SH-SY5Y cells.

## Discussion

In this study, we show that human TAU isoforms can influence microtubule dynamics in a differential matter, through i) differential compartment-specific cellular distribution and ii) differential effect on cell size and, to lesser extent by influencing microtubule density and microtubule dynamics. Expressed TAU isoforms with an N-terminal HA-tag differ in their subcellular localization in mouse primary neurons already after two days of expression. 2N-TAU isoforms especially showed less axonal enrichment than shorter isoforms, which is in line with previous findings, demonstrating less axonal enrichment for Dendra2c-tagged 2N-TAU isoforms (Zempel et al., 2017a). The shortest human TAU isoform, 0N3R, showed the strongest axonal enrichment compared with the other isoforms. Axonal sorting of endogenous mouse TAU was also highly efficient. During this stage of maturation (E13.5), mouse primary neurons mainly express 0N3R-TAU (McMillan et al., 2008; Bullmann et al., 2009), which supports our results obtained in this study demonstrating efficient sorting of human 0N3R-TAU in primary neurons. In contrast, especially 2N-TAU isoforms poorly localized to the axon. Since 2N3R and 1N4R nearly have the same size, with 410 and 412 aa, respectively, this effect is rather dependent on the N-terminal inserts and is independent of the corresponding isoform size. No significant difference was observed between the other 3R and 4R isoform-expressing neurons, pointing towards a repeat-independent sorting mechanism of the isoforms. Nevertheless, the C-terminal half of TAU is essential for proper axonal sorting, as a TAU construct lacking the repeat domains accumulates in the soma and did not localize to the axon in SH-SY5Y-derived and primary mouse neurons (Bell et al., 2021). These observations indicate that microtubule-binding affinity mediated by the C-terminal repeat domains [which is increased for four-repeat TAU isoforms (Goode et al., 2000)] alone does not mediate axonal sorting. Axonal sorting of TAU isoforms might also be influenced by PTMs, such as phosphorylation and acetylation, which are known to affect microtubule affinity and spacing (Evans et al., 2000; Kishi et al., 2005; Tsushima et al., 2015). In addition, isoform-specific protein interactions might influence axonal targeting (Gauthier-Kemper et al., 2018); however, this has not been addressed in detail for the different isoforms. Of note, axonal sorting of endogenous mouse TAU is more efficient compared to all HA-tagged human TAU isoforms. To rule out a saturation effect of the sorting machinery, we correlated TAU expression levels among our experimental groups with the axonal enrichment and found no significant effect. These results indicate that axonal sorting is independent of TAU expression levels and did not result in saturation of the sorting machinery of primary neurons. Since we could not observe the expected strong overexpression levels for pan-TAU in transfected neurons compared with neurons only expressing endogenous TAU (as reported before, e.g., in Tashiro et al., 1997), exogenous expression of TAU^HA^ likely is rather minor, or results in downregulation of endogenous TAU expression, preventing detrimental effects of overexpression.

The differences observed in axonal sorting of TAU isoforms might also have methodological reasons: i) Expression time was relatively short in our study, and increasing it from two to four days might be beneficial for the axonal enrichment of exogenous TAU. ii) The HA-tag might impair efficient axonal targeting of endogenous TAU, as seen also for Dendra2c-tagged TAU (Zempel et al., 2017a), due to its molecular size or by interfering with critical TAU interactions. iii) Sorting of human TAU might be different from mouse TAU, which could have several reasons: Young primary neurons, aged roughly one week, mainly express 0N3R-TAU; mouse TAU in general is slightly shorter; and the protein lifetime might be different due to tagging or changed protein interactions (Goedert et al., 1989; Kosik et al., 1989). In addition, the different TAU isoforms could influence each other and their sorting *in vivo*, while in this study, we analyzed the isoforms in an isolated approach. Overall, we show here that in polarized primary neurons, human TAU isoforms differ remarkably in their axonal sorting efficiencies (with 0N3R-TAU sorting ∼1.5 times better than the 2N-TAU isoforms), and that the somatic retention of 2N-TAU isoforms is repeat domain independent.

So far, no studies were conducted to investigate TAU isoform-specific effects on microtubule dynamics in a cellular context. We decided to use the well-established SH-SY5Y neuroblastoma cell line, which expresses only low levels of mainly 0N3R-TAU in the undifferentiated state (reviewed in Bell and Zempel, 2020) to investigate microtubule dynamics in dependence of the six different human TAU isoforms. 4R-TAU isoform-expressing cells showed a significant reduction in cell size (∼30%); however, microtubule numbers were slightly increased in these cells. These results go in line with *in vitro* studies showing an increased binding affinity and assembly of microtubules by 4R-TAU isoforms (Goedert and Jakes, 1990; Goode et al., 2000; Panda et al., 2003). Since cell size is affected in undifferentiated SH-SY5Y cells, there might be also an isoform-specific effect on neurite growth, which needs to be investigated in differentiated neuronal models. Microtubule dynamics were not significantly altered by the expression of individual TAU isoforms in our model system; however, there might be a compartment-specific effect visible in neurons, due to the differential localization of TAU isoforms demonstrated in the first part of this study. Possible compartment-specific effects might be caused by different PTMs, differences in microtubule-binding affinity, and different protein–protein interactions of TAU isoforms (Tashiro et al., 1997; Evans et al., 2000; Kishi et al., 2005; Tsushima et al., 2015). Of note, our results suggest that the N-terminal inserts play a major role in subcellular localization of TAU and, thus, may be important for the development or prevention of pathological processes, such as missorting of TAU into the somatodendritic compartment and subsequent synapse loss. This is in line with the observation that the presence of the H2 haplotype of the *MAPT* locus, resulting in a two times higher expression of exon 3 + transcripts (2N-TAU isoforms, respectively), has neuroprotective effects (Caffrey et al., 2006; Beevers et al., 2017). 2N-TAU isoforms were also shown to differ in their interactome from the other TAU isoforms, suggesting differences in the cellular functions of the TAU isoforms (Liu et al., 2016). While the generation and effects of the 3R- and 4R-TAU isoforms have been studied in detail, the splicing and behavior of the N-terminally different isoforms are understudied. Future studies with polarized cells, such as primary and iPSC-derived neurons, should aim to further characterize TAU isoforms regarding their impact on microtubule dynamics in different subcellular compartments, taking into account the differential sorting, impact on cell size, and microtubule number. In sum, we show that i) the efficiency of human TAU sorting into the axon is isoform dependent, with 2N isoforms being most retained in the somatodendritic compartment, and ii) 4R-TAU isoforms result in a general reduction of cell size and seem to increase microtubule counts. This points to isoform- and compartment-specific functions of TAU in neurons. Follow-up experiments are needed to clarify how the different TAU isoforms might influence cellular functions and if they contribute differentially to the pathological processes underlying AD and related tauopathies.

## Data Availability Statement

The original contributions presented in the study are included in the article/supplementary material, further inquiries can be directed to the corresponding author.

## Ethics Statement

Primary mouse neuron culture generation was reviewed and approved by Carolin Debuschewitz, Animal Welfare Officer of the University of Cologne (according to §4 Tierschutzgesetz).

## Author Contributions

SB: study design, data acquisition, analysis, interpretation, and drafting of the manuscript. MB and JK: assistance in data acquisition and methodology development. MB: manuscript proofreading. HZ: project funding, providing of concept, study design, interpretation of data, and drafting of the manuscript. All authors contributed to the article and approved the submitted version.

## Conflict of Interest

The authors declare that the research was conducted in the absence of any commercial or financial relationships that could be construed as a potential conflict of interest.
